# 2-(2-Pyridylsulfan­yl)acetic acid

**DOI:** 10.1107/S1600536809054373

**Published:** 2009-12-24

**Authors:** Xiao-Feng Li, Yan An, Hui-Guo Chen, Li-Hua Dong, Wei Yan

**Affiliations:** a Institute of Marine Materials Science and Engineering, Shanghai Maritime University, Shanghai 201306, People’s Republic of China.

## Abstract

All non-H atoms of the title compound, C_7_H_7_NO_2_S, lie on a crystallographic mirror plane, with the two methyl­ene H atoms bis­ected by this plane. The crystal packing is characterized by inter­molecular C—H⋯O and O—H⋯N contacts, which link the mol­ecules into infinite zigzag chains parallel to [010].

## Related literature

For background to the design of similar ligands, see: Akrivos (2001 ▶); Ye *et al.* (2005 ▶). For bond-length data, see: Allen *et al.* (1987 ▶).
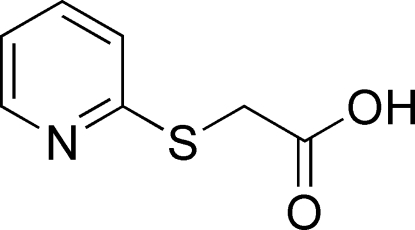


## Experimental

### 

#### Crystal data

C_7_H_7_NO_2_S
*M* *_r_* = 169.20
Orthorhombic, 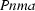

*a* = 14.5521 (19) Å
*b* = 6.6774 (13) Å
*c* = 7.7212 (19) Å
*V* = 750.3 (3) Å^3^
*Z* = 4
Mo *K*α radiation
μ = 0.37 mm^−1^
*T* = 293 K
0.37 × 0.35 × 0.27 mm

#### Data collection

Bruker APEXII CCD diffractometer
Absorption correction: multi-scan (*SADABS*; Sheldrick, 2004 ▶) *T*_min_ = 0.875, *T*_max_ = 0.906
1160 measured reflections
805 independent reflections
473 reflections with *I* > 2σ(*I*)
*R*_int_ = 0.066

#### Refinement

*R*[*F* ^2^ > 2σ(*F* ^2^)] = 0.050
*wR*(*F* ^2^) = 0.140
*S* = 1.00
805 reflections
67 parameters
H-atom parameters constrained
Δρ_max_ = 0.27 e Å^−3^
Δρ_min_ = −0.35 e Å^−3^

### 

Data collection: *APEX2* (Bruker, 2004 ▶); cell refinement: *SAINT* (Bruker, 2004 ▶); data reduction: *SAINT*; program(s) used to solve structure: *SHELXS97* (Sheldrick, 2008 ▶); program(s) used to refine structure: *SHELXL97* (Sheldrick, 2008 ▶); molecular graphics: *SHELXTL* (Sheldrick, 2008 ▶); software used to prepare material for publication: *SHELXTL*.

## Supplementary Material

Crystal structure: contains datablocks I, global. DOI: 10.1107/S1600536809054373/sj2712sup1.cif

Structure factors: contains datablocks I. DOI: 10.1107/S1600536809054373/sj2712Isup2.hkl

Additional supplementary materials:  crystallographic information; 3D view; checkCIF report

## Figures and Tables

**Table 1:** Hydrogen-bond geometry (Å, °)

*D*—H⋯*A*	*D*—H	H⋯*A*	*D*⋯*A*	*D*—H⋯*A*
O2—H2*B*⋯N1^i^	0.82	1.79	2.606 (5)	175
C2—H2*A*⋯O2^ii^	0.93	2.50	3.410 (6)	167
C3—H3*A*⋯O1^iii^	0.93	2.46	3.229 (5)	140

Symmetry codes: (i) 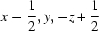; (ii) 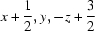; (iii) 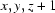.

**Fig. 1.**
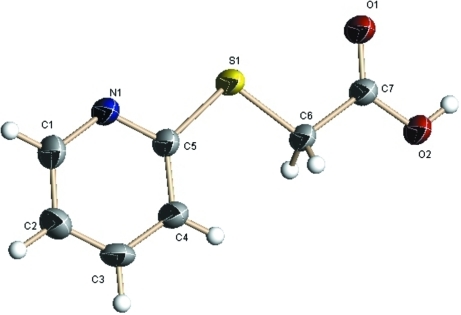
View of the structure of I. Displacement ellipsoids are drawn at the 30% probability level.

**Fig. 2.**
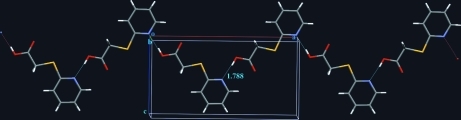
Crystal packin of the title compound viewed down the b axis.

## supplementary crystallographic information

### Comment

Compounds involving heterocyclic thiolate groups are ambidentate ligands which can form various metal-organic coordination structures *via* coordination of the exocyclic sulfur or the endocyclic nitrogen atoms (Akrivos, 2001). Similarly, carboxylic acids also exhibit diverse coordination modes in different metal complexes (Ye *et al.*, 2005). In attempts to develop novel coordination frameworks, we  have designed and synthesized the title compound, 2-(pyridin-2-ylthio)acetic acid (I), as a potentially multidentate ligand. Its crystal structure is reported here.

The single-crystal X-ray analysis of I reveals that all the bond lengths in compound I are within normal ranges (Allen *et al.*, 1987). All the non-hydrogen atoms in each molecule are coplanar with the methylene hydrogen atoms related by mirror symmetry (Fig. 1). In the crystal structure molecules are linked into infinite, one dimensional, zigzag chains due to intermolecular H-bonding (Fig. 2, Table 2).

### Experimental

The title compound was prepared by heating a mixture of 2-pyridinethione (0.335 g, 3 mmol), chloroacetic acid (0.292 g, 3.1 mmol) and sodium hydroxide (0.248 g, 6.2 mmol) in ethanol at 353 K with magnetic stirring for 8 h. The pH of the solution was adjusted to 6 with hydrochloric acid. Yellow crystals were obtained after being recrystrallized twice from the ethanol solution (yield 78%). Analysis, calculated for C_7_H_7_NO_2_S: C 49.69, H 4.17, N 8.28%; Found: C 50.06, H 4.27, N 8.06%.

### Refinement

All H-atoms were positioned geometrically and refined using a riding model with d(C-H) = 0.93Å,  U_iso_=1.2U_eq_ (C) for aromatic 0.97Å,  U_iso_ = 1.2U_eq_ (C) for CH_2_, and 0.82Å, U_iso_ = 1.5U_eq_ (O) for the OH group.

### Crystal data

**Table:**

C_7_H_7_NO_2_S	*F*(000) = 352
*M**_r_* = 169.20	*D*_x_ = 1.498 Mg m^−^^3^
Orthorhombic, *P**n**m**a*	Mo *K*α radiation, λ = 0.71073 Å
Hall symbol: -P 2ac 2n	Cell parameters from 343 reflections
*a* = 14.5521 (19) Å	θ = 2.8–28.0°
*b* = 6.6774 (13) Å	µ = 0.37 mm^−^^1^
*c* = 7.7212 (19) Å	*T* = 293 K
*V* = 750.3 (3) Å^3^	Block, yellow
*Z* = 4	0.37 × 0.35 × 0.27 mm

### Data collection

**Table:**

Bruker APEXII CCD diffractometer	805 independent reflections
Radiation source: fine-focus sealed tube	473 reflections with *I* > 2σ(*I*)
graphite	*R*_int_ = 0.066
φ and ω scans	θ_max_ = 26.0°, θ_min_ = 2.8°
Absorption correction: multi-scan (*SADABS*; Sheldrick, 2004)	*h* = −17→1
*T*_min_ = 0.875, *T*_max_ = 0.906	*k* = −1→8
1160 measured reflections	*l* = −9→1

### Refinement

**Table:**

Refinement on *F*^2^	Primary atom site location: structure-invariant direct methods
Least-squares matrix: full	Secondary atom site location: difference Fourier map
*R*[*F*^2^ > 2σ(*F*^2^)] = 0.050	Hydrogen site location: inferred from neighbouring sites
*wR*(*F*^2^) = 0.140	H-atom parameters constrained
*S* = 1.00	*w* = 1/[σ^2^(*F*_o_^2^) + (0.0634*P*)^2^] where *P* = (*F*_o_^2^ + 2*F*_c_^2^)/3
805 reflections	(Δ/σ)_max_ = 0.003
67 parameters	Δρ_max_ = 0.27 e Å^−^^3^
0 restraints	Δρ_min_ = −0.35 e Å^−^^3^

### Special details

**Table:**

Geometry. All e.s.d.'s (except the e.s.d. in the dihedral angle between two l.s. planes) are estimated using the full covariance matrix. The cell e.s.d.'s are taken into account individually in the estimation of e.s.d.'s in distances, angles and torsion angles; correlations between e.s.d.'s in cell parameters are only used when they are defined by crystal symmetry. An approximate (isotropic) treatment of cell e.s.d.'s is used for estimating e.s.d.'s involving l.s. planes.
Refinement. Refinement of *F*^2^ against ALL reflections. The weighted *R*-factor *wR* and goodness of fit *S* are based on *F*^2^, conventional *R*-factors *R* are based on *F*, with *F* set to zero for negative *F*^2^. The threshold expression of *F*^2^ > σ(*F*^2^) is used only for calculating *R*-factors(gt) *etc*. and is not relevant to the choice of reflections for refinement. *R*-factors based on *F*^2^ are statistically about twice as large as those based on *F*, and *R*- factors based on ALL data will be even larger.

### Fractional atomic coordinates and isotropic or equivalent isotropic displacement parameters (Å^2^)

**Table:**

	*x*	*y*	*z*	*U*_iso_*/*U*_eq_	Occ. (<1)
S1	0.34558 (7)	0.2500	0.33858 (14)	0.0562 (6)	
C1	0.5342 (3)	0.2500	0.6916 (6)	0.0583 (19)	
H1A	0.5981	0.2500	0.6879	0.070*	
C2	0.4919 (3)	0.2500	0.8503 (6)	0.0560 (17)	
H2A	0.5262	0.2500	0.9520	0.067*	
C3	0.3973 (3)	0.2500	0.8547 (6)	0.0545 (17)	
H3A	0.3668	0.2500	0.9605	0.065*	
C4	0.3481 (3)	0.2500	0.7029 (5)	0.0484 (15)	
H4A	0.2842	0.2500	0.7050	0.058*	
C5	0.3950 (3)	0.2500	0.5463 (5)	0.0452 (15)	
C6	0.2251 (2)	0.2500	0.3861 (5)	0.0459 (15)	
H6A	0.2093	0.1322	0.4533	0.055*	0.50
H6B	0.2093	0.3678	0.4533	0.055*	0.50
C7	0.1723 (3)	0.2500	0.2160 (6)	0.0454 (15)	
N1	0.4877 (2)	0.2500	0.5405 (5)	0.0474 (13)	
O1	0.2095 (2)	0.2500	0.0775 (4)	0.0638 (13)	
O2	0.08419 (18)	0.2500	0.2437 (4)	0.0557 (12)	
H2B	0.0569	0.2500	0.1508	0.084*	

### Atomic displacement parameters (Å^2^)

**Table:**

	*U*^11^	*U*^22^	*U*^33^	*U*^12^	*U*^13^	*U*^23^
S1	0.0240 (6)	0.1177 (15)	0.0269 (6)	0.000	−0.0001 (5)	0.000
C1	0.025 (2)	0.109 (6)	0.041 (3)	0.000	−0.0074 (19)	0.000
C2	0.042 (3)	0.095 (5)	0.031 (2)	0.000	−0.007 (2)	0.000
C3	0.041 (3)	0.099 (5)	0.023 (2)	0.000	0.006 (2)	0.000
C4	0.028 (2)	0.086 (5)	0.031 (2)	0.000	0.0029 (18)	0.000
C5	0.023 (2)	0.083 (5)	0.030 (2)	0.000	−0.0021 (17)	0.000
C6	0.0196 (19)	0.088 (5)	0.030 (2)	0.000	−0.0003 (17)	0.000
C7	0.027 (2)	0.080 (5)	0.029 (2)	0.000	0.0009 (18)	0.000
N1	0.0240 (17)	0.088 (4)	0.0304 (18)	0.000	−0.0001 (15)	0.000
O1	0.0268 (15)	0.134 (4)	0.0310 (16)	0.000	−0.0006 (13)	0.000
O2	0.0211 (16)	0.112 (4)	0.0345 (16)	0.000	−0.0018 (13)	0.000

### Geometric parameters (Å, °)

**Table:**

S1—C5	1.757 (4)	C4—C5	1.389 (6)
S1—C6	1.791 (4)	C4—H4A	0.9300
C1—N1	1.348 (5)	C5—N1	1.350 (5)
C1—C2	1.372 (6)	C6—C7	1.522 (6)
C1—H1A	0.9300	C6—H6A	0.9700
C2—C3	1.377 (6)	C6—H6B	0.9700
C2—H2A	0.9300	C7—O1	1.198 (5)
C3—C4	1.374 (6)	C7—O2	1.301 (5)
C3—H3A	0.9300	O2—H2B	0.8200
			
C5—S1—C6	102.31 (19)	N1—C5—S1	112.3 (3)
N1—C1—C2	123.2 (4)	C4—C5—S1	126.4 (3)
N1—C1—H1A	118.4	C7—C6—S1	108.5 (3)
C2—C1—H1A	118.4	C7—C6—H6A	110.0
C1—C2—C3	118.1 (4)	S1—C6—H6A	110.0
C1—C2—H2A	121.0	C7—C6—H6B	110.0
C3—C2—H2A	121.0	S1—C6—H6B	110.0
C4—C3—C2	120.0 (4)	H6A—C6—H6B	108.4
C4—C3—H3A	120.0	O1—C7—O2	126.3 (4)
C2—C3—H3A	120.0	O1—C7—C6	122.9 (4)
C3—C4—C5	119.1 (4)	O2—C7—C6	110.8 (4)
C3—C4—H4A	120.4	C1—N1—C5	118.3 (4)
C5—C4—H4A	120.4	C7—O2—H2B	109.5
N1—C5—C4	121.3 (4)		
			
N1—C1—C2—C3	0.000 (1)	C5—S1—C6—C7	180.0
C1—C2—C3—C4	0.000 (1)	S1—C6—C7—O1	0.0
C2—C3—C4—C5	0.000 (1)	S1—C6—C7—O2	180.0
C3—C4—C5—N1	0.0	C2—C1—N1—C5	0.0
C3—C4—C5—S1	180.0	C4—C5—N1—C1	0.0
C6—S1—C5—N1	180.0	S1—C5—N1—C1	180.0
C6—S1—C5—C4	0.0		

### Hydrogen-bond geometry (Å, °)

**Table:**

*D*—H···*A*	*D*—H	H···*A*	*D*···*A*	*D*—H···*A*
O2—H2B···N1^i^	0.82	1.79	2.606 (5)	175
C2—H2A···O2^ii^	0.93	2.50	3.410 (6)	167
C3—H3A···O1^iii^	0.93	2.46	3.229 (5)	140

Symmetry codes: (i) *x*−1/2, *y*, −*z*+1/2; (ii) *x*+1/2, *y*, −*z*+3/2; (iii) *x*, *y*, *z*+1.
